# Higher helminth ova counts and incomplete decomposition in sand-enveloped latrine pits in a coastal sub-district of Bangladesh

**DOI:** 10.1371/journal.pntd.0010495

**Published:** 2022-06-23

**Authors:** Mahbubur Rahman, Mahfuza Islam, Solaiman Doza, Abu Mohammed Naser, Abul Kasham Shoab, Julia Rosenbaum, Md. Shariful Islam, Leanne Unicomb, Thomas F. Clasen, Ayse Ercumen

**Affiliations:** 1 Environmental Intervention Unit, Infectious Disease Division, icddr,b, Dhaka, Bangladesh; 2 Gangarosa Department of Environmental Health Sciences, Rollins School of Public Health, Emory University, Atlanta, Georgia, United States of America; 3 Division of Epidemiology, Biostatistics, and Environmental Health, School of Public Health, University of Memphis, Tennessee, United States of America; 4 FHI360, Washington DC, United States of America; 5 Department of Agricultural Chemistry, Patuakhali Science and Technology University, Patuakhali, Bangladesh; 6 Department of Forestry and Environmental Resources, North Carolina State University, Raleigh, North Carolina, United States of America; California Department of Public Health, UNITED STATES

## Abstract

Pit latrines are the most common latrine technology in rural Bangladesh, and untreated effluent from pits can directly contaminate surrounding aquifers. Sand barriers installed around the latrine pit can help reduce contamination but can also alter the decomposition of the fecal sludge and accelerate pit fill-up, which can counteract their benefits. We aimed to evaluate whether there was a difference in decomposition of fecal sludge and survival of soil-transmitted helminth (STH) ova among latrines where a 50-cm sand barrier was installed surrounding and at the bottom of the pit, compared to latrines without a sand barrier, in coastal Bangladesh. We assessed decomposition in latrine pits by measuring the carbon-nitrogen (C/N) ratio of fecal sludge. We enumerated *Ascaris lumbricoides* and *Trichuris trichiura* ova in the pit following 18 and 24 months of latrine use. We compared these outcomes between latrines with and without sand barriers using generalized linear models with robust standard errors to adjust for clustering at the village level. The C/N ratio in latrines with and without a sand barrier was 13.47 vs. 22.64 (mean difference: 9.16, 95% CI: 0.15, 18.18). Pits with sand barriers filled more quickly and were reportedly emptied three times more frequently than pits without; 27/34 latrines with sand barriers vs. 9/34 latrines without barriers were emptied in the previous six months. Most reported disposal methods were unsafe. Compared to latrines without sand barriers, latrines with sand barriers had significantly higher log_10_ mean counts of non-larvated *A*. *lumbricoides* ova (log_10_ mean difference: 0.35, 95% CI: 0.12, 0.58) and *T*. *trichiura* ova (log_10_ mean difference: 0.47, 95% CI: 0.20, 0.73). Larvated ova counts were similar for the two types of latrines for both *A*. *lumbricoides* and *T*. *trichiura*. Our findings suggest that sand barriers help contain helminth ova within the pits but pits with barriers fill up more quickly, leading to more frequent emptying of insufficiently decomposed fecal sludge. Further research is required on latrine technologies that can both isolate pathogens from the environment and achieve rapid decomposition.

## Author summary

Conventional pit latrines do not fully isolate fecal pathogens and in many settings, pits are emptied manually, and pit contents released into water bodies and fields. Sand barriers installed around the latrine pit can help reduce contamination but can also alter the decomposition of the fecal sludge. We evaluated whether a sand barrier effectively contains STH ova within the pit and whether it alters the decomposition processes of pit contents and/or accelerates pit fill-up. C/N ratio was significantly higher in latrines without a sand barrier and approached the preferable range for optimal decomposition. Pit latrines with sand barriers filled up more rapidly and were emptied more often. No households in either arm used any protective measure when emptying the pits. Larvated STH ova counts were similar in the latrines with vs. without sand barriers while non-larvated ova counts were higher in pits with a sand barrier. The lower C/N ratio and quicker filling up of latrines with sand barriers indicate that pit contents are more likely to be infective at the time of pit emptying, necessitating safe emptying and disposal methods. Latrines with and without sand barrier had similar loading into the pit by infected individuals. Enveloping latrine pits with a sand layer helped contain helminth ova within the pits, allowing time for them to become non-viable and potentially reducing the spread of viable ova into the surrounding environment.

## Introduction

Improved sanitation is the primary barrier to prevent fecal contamination from entering the environment. Pit latrines are one of the most commonly used human excreta disposal systems in low-income countries due to their low cost and availability [1]. Globally, an estimated 1.8 billion people use pit latrines as the primary means of sanitation [2], and construction of pit latrines is increasing as countries strive to meet the Sustainable Development Goals on sanitation [3]. Pit latrines are the most common latrine technology in rural Bangladesh. However, conventional pit latrines do not fully isolate fecal pathogens. Viruses and bacteria, and to a smaller extent protozoa, can infiltrate from pit latrines into surrounding soils and aquifers [2, 4, 5]. While recommendations exist for safe management of fecal sludge from on-site sanitation facilities (i.e., sludge emptying, transport, disposal) [6], in many settings, pits are emptied manually and pit contents released into water bodies and fields [7, 8], including nearby ponds and agricultural soils. This practice can further spread fecal pathogens if pits are emptied before decomposition and pathogen inactivation is complete. Pathogens from these environmental reservoirs can be tracked into the living environment via humans, domestic animals, and flies, where they can contaminate stored drinking water, food, hands, surfaces, and objects.

Soil-transmitted helminth (STH) infections affect more than 1.5 billion people worldwide [9]. These infections include *Ascaris lumbricoides* (roundworm), *Trichuris trichiura* (whipworm) and hookworms. Sanitation systems that isolate human feces from the environment should prevent potential new hosts from ingesting STH ova from the feces of infected individuals. Meta-analyses have suggested that sanitation improvements are associated with reduced risk of STH infection [10, 11]. However, there are little empirical data on the impact of sanitation interventions on STH ova measured directly in the environment. Two recent randomized trials in rural Bangladesh and Kenya found no reduction in STH ova in courtyard soil among recipients of onsite pit latrines [12, 13]. One study in urban Mozambique evaluated the impact of a shared onsite sanitation intervention on enteric in soil at the latrine entrance. *A*. *lumbricoides* was the most commonly detected pathogen, and the latrine intervention significantly reduced its prevalence in soil, measured 24 months after intervention implementation [14]. Installing sand-barriers around latrine pits is hypothesized to reduce STH transmission by reducing the prevalence and/or concentration of STH ova in the environment surrounding the latrine pit.

A sand barrier can reduce leaching of pathogens from pit latrines by providing a mechanism for filtration and biological removal [15]. One important biological mechanisms is the formation of a “biomat” (layer of microorganisms) on the sand surface using air within the sand barrier and nutrients from the pit contents [16]. We previously conducted a double-arm randomized controlled trial in Galachipa, a coastal sub-district of Bangladesh with a high ground water table, to test an improved latrine design that included a 50-cm sand barrier surrounding and at the bottom of the pit [15]. This study found that pit latrines with a sand barrier had significantly reduced leaching of *E*. *coli* and thermotolerant coliforms into ground water, especially during the dry months, compared to pit latrines without a sand barrier [15].

Installing sand barriers can also affect how quickly decomposition occurs in latrine pits and how frequently they need to be emptied. Two types of decomposition of pit contents occur under normal circumstances: aerobic and anaerobic decomposition. Aerobic decomposition occurs close to the surface of the pit, where contents make contact with air, while anaerobic decomposition occurs in the remaining parts of the pit [17]. During the decomposition process, pathogens are inactivated, sludge volume decreases, and the chemical and biological properties of the sludge change [17]. Aerobic decomposition is faster than anaerobic decomposition and more efficiently inactivates pathogens as it generates more heat [17]. However, aerobic decomposition does not reduce sludge volume as much as anaerobic decomposition [17]. We hypothesized that installing a sand barrier may favor more aerobic decomposition due to more air within the sand layer which is more porous than soil [18]. It is therefore possible that, while pit latrines with a sand barrier have quicker decomposition, they can also fill up more quickly and need to be emptied more often, with less time for inactivation of pathogens.

In rural Bangladesh, filled pits are often emptied manually using buckets and ropes without safety precautions [8], and the contents are released into waterways or fields, posing a risk for water and soil contamination and directly to humans emptying the pits [19]. Often, pits are emptied without adequate time for pathogen inactivation due to rapid fill-up. In this study, we aimed to evaluate whether a sand barrier effectively contains STH ova within the pit and whether it alters the decomposition processes of pit contents and/or accelerates pit fill-up.

## Methods

### Ethics statement

Selected households were informed about our study objectives and their right to discontinue participation at any point of the study period. Informed written consent was taken from all household heads prior to installing the latrines. The study protocol was reviewed and approved by the human subjects’ review committee at International Centre for Diarrhoeal Disease and Research, Bangladesh (icddr, b) (PR-14117).

### Study design and setting

We conducted a randomized controlled trial from December 2015 to May 2016 in Galachipa sub-district of coastal Bangladesh embedded within the USAID/WASH plus project. The project was implemented through FHI 360 and WaterAid through local NGOs that identified extremely poor households in the area, termed as “hardcore poor” on government social inventories. The implementing organizations selected 68 households that met the eligibility criteria and constructed low-cost latrines. Eligibility criteria were: (i) household had 4–10 members (to ensure standard pit loading rate), (ii) land was available to construct new pit latrines at least 5 meters away from existing unimproved latrines, and (iii) the land donated by the household for pit latrine construction was not adjacent to surface water bodies.

We block randomized the 68 households into two groups, one group (intervention) to receive new pit latrines with a sand barrier and one group (control) to receive pit latrines without a sand barrier. A statistician was responsible for generating a unique household ID that included the randomization assignment and a sealed envelope that was coded for latrines with and without sand barriers. The codes were only shared with construction crews responsible for latrine installation. Additional details of the randomized trial design have been described elsewhere [15].

### Latrine and sand barrier construction

For the construction of latrines and sand barriers, the study team recruited three local contractors and supervised the construction closely to ensure structure specifications were met (S1 Text). The latrines were constructed with five concrete liner rings of 300 mm height for the pit. The contractors used locally available sand to build a 50-cm sand barrier around and below the concrete rings for the latrines with sand barriers [15]. Cost of construction was USD 282 for latrines with sand barriers and USD 257 for those without.

### Promotion of latrine use

The study team recruited and trained eleven community health promoters who were uninformed of the study objectives or methods. The promoters delivered messages on use, maintenance, and safe emptying of latrines, and instructed households to demolish any previously constructed latrines.

### Data collection

The randomized control trial conducted follow-up visits at 12, 15, 18 and 24 months after installation of new latrines. In this study, we collected additional data and samples from the 68 households that were participating in the ongoing trial at the 18-month and 24-month follow-up visits. To determine reported pit-emptying practices, field staff administered a short survey at both follow-ups and recorded whether the emptied sludge was buried or disposed into a surface water body (river/canal/pond/ditches). At each visit, field staff also collected sludge samples from the latrines to enumerate larvated and non-larvated STH ova and to measure the pH, temperature, moisture content, and carbon/nitrogen (C/N) ratio. Larvated STH ova indicate viable organisms shed into the latrine by infected household members using the latrine while non-larvated ova indicate inactivated organisms. High temperature is suggestive of aerobic decomposition, high pH is recommended for pathogen inactivation, and low moisture and high C/N ratio are suggestive of more complete decomposition [17], with C/N ratios ranging from 25–35 indicating adequate decomposition [17].

### Sample collection and processing

Field staff collected four separate fecal sludge samples of 200 g each from each pit using a 2-m long stainless-steel T-shaped scoop. Before collecting the samples, the scoop was first washed with distilled water, then washed with 10% bleach and again washed with distilled water and then wiped dry with a tissue paper. Similar steps were repeated after collecting the samples. The scoop was inserted through the top layers of the pit down to 1 m deep and pushed toward the pit wall to allow the sludge to enter through the open end. The closed end of the scoop had multiple small holes to discard the excess fluid. The collected samples were placed in an airtight sterile Whirl-Pak bag (Nasco Modesto, Salida, CA) and transported to the laboratory of Agro-analytical Chemistry, Department of Agricultural Chemistry, Patuakhali Science and Technology University in a cooler box maintaining 2–8°C.

Moisture content and pH were measured using *Standard Methods for the Examination of Water and Wastewater* [20]. The temperature of the sludge was measured onsite using a compost thermometer (REOTEMP). To estimate the C/N ratio, the organic carbon content was determined by the wet combustion (Walkley-Black method) technique [19], and total nitrogen content was estimated using the Kjeldahl method [21]. A sludge sample aliquot of 200 g was sent to the field laboratory of the International Centre for Diarrhoeal Disease Research, Bangladesh (icddr,b) to quantify ova of *A*. *lumbricoides* and *T*. *trichiura* using a protocol adapted from the USEPA method for enumerating *Ascaris* ova in fecal sludge [22, 23]. In laboratory experiments, the adapted protocol demonstrated a recovery efficiency of 73% for *Ascaris suum* ova, which are morphologically identical to *A*. *lumbricoides* [23]. As the method was optimized to detect *A*. *lumbricoides*, the recovery efficiency for other STH species was likely lower, and previous applications of the method did not detect hookworm ova in soil samples in Bangladesh and Kenya [12, 13, 23]. We therefore did not aim to enumerate hookworm ova in our study.

In brief, a 15 g fecal sludge aliquot was soaked overnight in 1% 7X detergent solution, then hand-shaken for 10 minutes and vortexed on 2000 rpm for 15 seconds to dislodge STH ova from particles. The solution was poured through a 50-mesh sieve to remove large particles. The supernatant was left to settle for 2 hours and then aspirated without disturbing the residue at the bottom. Approximately 40 mL of 1% 7X solution was added to the precipitate and the solution was centrifuged at 1000 g for 10 minutes; the supernatant was discarded. Next, 5 mL of zinc sulphate flotation solution (1.25 specific gravity) was added to the precipitate, vortexed for 30 seconds, centrifuged at 1000 g for 5 minutes and the supernatant was saved; this procedure was conducted a total of three times. The combined supernatant from the three flotation steps was filtered through a 500-mesh sieve to capture STH ova. The sieve was rinsed into a Falcon tube using distilled water, the rinse water was centrifuged at 1000 g for 5 min, and the supernatant was removed with a pipette until there was 1 mL left at the bottom of the tube. Next, 25 mL of 0.1 N sulfuric acid solutions was added to the tube. The tube was capped loosely and incubated at 28° C for 28 days to allow viable ova to develop larvae. At the end of the incubation period, the solution was centrifuged at 1000 g for 3 min and aspirated to a final volume of 1 mL. The 1 mL solution was transferred to a Sedgewick-Rafter slide and examined under the microscope for *A*. *lumbricoides* and *T*. *trichiura* ova using a visual identification chart to distinguish the type of ova and whether it was larvated or non-larvated. The numbers of larvated and non-larvated ova for each species were recorded separately to differentiate viable and non-viable ova. An additional 5 g sludge aliquot was oven-dried overnight to determine moisture content and dry weight. For quality assurance and quality control, 10% of samples were processed in replicate, and a laboratory blank was processed once every other day by repeating the protocol without a sludge sample. 10% of samples were counted by two independent analysts to assess interrater reliability. Additionally, for each sample, lab technicians took a picture of the first occurrence of each type of ova (larvated *A*. *lumbricoides*, non-larvated *A*. *lumbricoides* etc.); the pictures were reviewed for accuracy of categorization by study investigators.

### Data analysis

We used generalized linear models to compare outcomes between latrines with and without sand barriers, with robust standard errors to adjust for clustering at the village level. We pooled data from both sampling time points and also conducted individual analyses for the 18-month and 24-months sampling points. For normally distributed data (temperature, pH, moisture content, and C/N ratio), we used the Gaussian family. For STH ova, we calculated geometric means to reduce the skewness of our original data by log_10_ transforming ova count per dry gram of sludge after replacing counts of 0 eggs per gram (epg) with 0.5 epg. We estimated the mean difference in geometric mean ova counts between latrines with and without sand barriers using negative binomial models.

## Results

Randomization balanced enrolment characteristics between households in the intervention and control arms (Table 1). Our previous analysis of data from this trial found that the average number of household members, pre-intervention latrine access, median distance from the newly installed study latrine to the nearest existing latrine, and presence of a surface water body within 10 m of the study latrines was similar between intervention and control households [15].

**Table 1:** Baseline characteristics of the intervention and control households.

Characteristics		Intervention N = 34	Control N = 34
		% (n)	% (n)
Education of household head			
	None or primary	94 (32)	94 (32)
	Secondary or above	6 (2)	6 (2)
Homestead land owned (decimal), mean (SD)		18 (13)	18 (17)
Farm land owned (decimal), mean (SD)		27 (36)	27 (37)
Household owns mobile		94 (32)	91 (31)
Households with pre-intervention latrine		76 (26)	79 (27)
Water source within 10 meters of the study latrine		6 (4)	9 (6)
Surface water body within 10 meters of the study latrine		50 (17)	56 (19)
Household’s primary source of drinking water			
	Deep tubewell^a^	65 (22)	76 (26)
	Shallow tubewell^b^	33 (11)	24 (8)

^a^**Deep tubewell** defined as tubewell with > = 250 feet depth

^b^**Shallow tube well** defined as tubewell with <250 feet depth

### Temperature, pH, moisture content and C/N ratio of latrine sludge

Latrines with and without sand barriers had the same temperature (97.4°F vs. 97.5°F), average pH (7.44), and moisture content (71.9% vs. 71.4%) (Table 2). The C/N ratio was 13.5 in latrines with a sand barrier vs. 22.6 in latrines without a sand barrier (mean difference: 9.16, 95% CI: 0.15, 18.18). The difference in C/N ratio between the two types of latrines was driven by the 24-month follow-up (mean difference: 17.98, 95% CI: 1.23, 34.73); the C/N ratio was similar for both latrine types at the 18-month follow-up (Table 2).

**Table 2:** Average temperature, pH, percentage of moisture content, and carbon-nitrogen ratio of latrine sludge samples at 18 and 24 months of follow-up.

	Follow-up	Mean (SD)		Mean difference^a^	95% CI
		Intervention N = 34	Control N = 34		
Temperature (°F)	18 months	98.0 (0.28)	98.0 (0.36)	0.03	-0.12, 0.18
	24 months	96.9 (0.34)	97.0 (0.35)	0.08	-0.08, 0.25
	Combined	97.4 (0.62)	97.5 (0.63)	0.06	-0.05, 0.17
pH	18 months	7.41 (0.15)	7.41 (0.15)	0.00	-0.07, 0.07
	24 months	7.47 (0.24)	7.48 (0.48)	0.01	-0.17, 0.19
	Combined	7.44 (0.20)	7.44 (0.36)	0.00	-0.09, 0.10
% Moisture content	18 months	73.3 (15.2)	71.5 (11.4)	-1.87	-8.27, 4.52
	24 months	70.5 (15.6)	71.3 (13.3)	0.77	-6.12, 7.66
	Combined	71.9 (15.4)	71.4 (12.4)	-0.55	-5.63, 4.53
C/N ratio	18 months	15.2 (10.1)	15.5 (10.8)	0.35	-4.61, 5.31
	24 months	11.8 (9.49)	29.8 (8.92)	**18.0**	**1.23, 34.7**
	Combined	13.5 (9.88)	22.6 (16.0)	**9.16**	**0.15, 18.2**

CI: Confidence Interval

^a^We determined the mean difference by using generalized linear models (glm) with robust standard errors.

### STH ova in latrine sludge

The log_10_ transformed mean STH ova count per dry gram of sludge in latrines with sand barriers was 3.08 for non-larvated *A*. *lumbricoides*, 2.21 for larvated *A*. *lumbricoides*, 2.13 for non-larvated *T*. *trichiura*, and 1.16 for larvated *T*. *trichiura* (Table 3). The log_10_ transformed mean STH ova count per dry gram of sludge in latrines without sand barriers was 2.73 for non-larvated *A*. *lumbricoides*, 1.97 for larvated *A*. *lumbricoides*, 1.67 for non-larvated *T*. *trichiura*, and 0.93 for larvated *T*. *trichiura* (Table 3). Compared to latrines without sand barriers, latrines with sand barriers had significantly higher count of non-larvated *A*. *lumbricoides* ova (log_10_ mean difference: 0.35, 95% CI: 0.12, 0.58) and non-larvated *T*. *trichiura* ova (log_10_ mean difference: 0.47, 95% CI: 0.20, 0.73) (Table 3). There were no statistically significant differences in larvated ova counts between the two types of latrines for *A*. *lumbricoides* and *T*. *trichiura* (Table 3). Both the non-larvated and larvated *T*. *trichiura* counts declined by more than 50% between the 18-month and 24-month follow-up (Table 3).

**Table 3:** Mean log_10_ helminth ova counts per dry gram of latrine sludge at 18 and 24 months of follow-up.

Log_10_ mean helminth count/dry gram sludge	Follow-up	Log_10_ mean (SD)		Log_10_ mean difference^a^	95% CI
		Intervention N = 34	Control N = 34		
Non-larvated *Ascaris lumbricoides*	18 months	3.05 (0.10)	2.70 (0.13)	**0.35**	**0.03, 0.67**
	24 months	3.12 (0.10)	2.76 (0.14)	**0.36**	**0.02, 0.69**
	Combined	3.08 (0.07)	2.73 (0.09)	**0.35**	**0.12, 0.58**
Larvated *Ascaris lumbricoides*	18 months	2.04 (0.15)	1.84 (0.13)	0.20	-0.19, 0.59
	24 months	2.38 (0.11)	2.10 (0.17)	0.29	-0.11, 0.68
	Combined	2.21 (0.10)	1.97 (0.10)	0.24	-0.05, 0.53
Non-larvated *Trichuris trichiura*	18 months	2.43 (0.11)	2.07 (0.15)	0.36	-0.01, 0.72
	24 months	1.84 (0.10)	1.27 (0.15)	**0.57**	**0.22, 0.92**
	Combined	2.13 (0.07)	1.67 (0.11)	**0.47**	**0.20, 0.73**
Larvated *Trichuris trichiura*	18 months	1.42 (0.11)	1.22 (0.14)	0.19	-0.15, 0.53
	24 months	0.89 (0.11)	0.70 (0.14)	0.25	-0.09, 0.60
	Combined	1.16 (0.08)	0.93 (0.09)	0.22	-0.04, 0.48

SD: Standard Deviation; CI: Confidence Interval

^a^We determined the log_10_ mean difference by using negative binomial models with robust standard errors.

### Pit-emptying practices

The average pit fill-up time was 4 months in the intervention arm and 7 months in the control arm (Table 4). Nine of the 34 pits with sand barriers and three of the 34 control pits without sand barriers were emptied since our last visit (within last 6 months) (Table 4). One latrine in each group was emptied more than once (3 times) during this period. In the intervention arm, seven households disposed sludge into a surface water body (river/canal/pond/ditches) and two households buried the sludge to prevent recontamination whereas all three households in the control arm disposed of the sludge in a surface water body (Table 4). No households in either arm used any protective measure when emptying the pits, and all pits were emptied manually using a bucket and shovel.

**Table 4:** Pit emptying practices of the intervention and control latrine households.

		Intervention N = 34	Control N = 34
		% (n)	% (n)
Pit emptied since last visit (within last 6 months)			
	At least once	27 (9)	9 (3)
	Multiple times	3 (1)	3 (1)
Average pit fill-up duration (months)		4	7
Pit emptied manually		27 (9)	9 (3)
No protective measure when emptying pit^a^		27 (9)	9 (3)
Sludge disposed			
	Buried	6 (2)	-
	Into surface water body (river/canal/pond/ditches)	21 (7)	9 (3)

^a^No protective measure defined as did not wear gloves, face mask, apron or shoes while disposing of sludge

## Discussion

We found no difference in pH, temperature, and moisture content between latrines with and without a sand barrier while latrines without a barrier had a significantly higher C/N ratio. A pH of 9 or greater is desired for aerobic decomposition and pathogen inactivation [17]. Temperatures >104°F are needed to inactivate pathogens within a 1-year storage time, and at higher temperatures (>122°F), pathogen inactivation proceeds rapidly [17, 24]. In addition, low levels of moisture (≤5%) are needed to inactivate *Ascaris* ova if no other pathogen removal mechanisms are employed [24]. In our study, latrines with and without sand barriers had similar average pH (7.4) and temperature (97.4°F), which were below the recommended values for optimal aerobic decomposition and pathogen inactivation. Latrines with and without sand barriers also had similar moisture content (71%), which was much higher than ideal for pathogen inactivation. The average C/N ratio of the pits without sand barriers was significantly higher than pits with sand barriers and approached the preferable range of 25–35 for optimal decomposition, indicating that pits without the barrier went through more complete decomposition. Despite the favorable C/N ratio, pits without sand barriers contained viable STH ova. As we collected sludge from a depth of 1m, we expect that viable ova detected in the pit indicate prolonged survival rather than recent shedding by infected household members.

While latrines with sand barriers reduced the leaching of *E*. *coli* and thermotolerant coliforms into groundwater in our previous assessment [15], in the present analysis, we found that pit latrines with sand barriers also filled up more rapidly and were emptied more often than latrines without. The rapid fill-up suggests that the sand barriers enabled aerobic decomposition which does not reduce sludge volume as much as anaerobic decomposition. Pit-emptying practices were poor for both types of latrines. Most pits were emptied manually without any protective measures, and the sludge was disposed of in rivers, canals, and ditches. Taken together, the lower C/N ratio and quicker filling up of latrines with sand barriers indicate that pit contents are more likely to be infective at the time of pit emptying, necessitating safe emptying and disposal methods. A study conducted in rural El Salvador found that burying the fecal sludge from latrines compared to using it on household plants and trees was associated with lower prevalence of helminth and protozoa infection among households members [25]. This study also found that transmission of helminths was more likely to occur during emptying of the latrine compared to contact with the sludge after it was buried [25]. In settings where pit latrines with sand barriers are installed to reduce leaching of pathogens from pits, safe pit emptying, and sludge disposal practices need to be emphasized to reduce risks from incomplete decomposition. Alternatively, double-pit latrines can be installed to alternate between pits and allow time for decomposition of pit contents before they need to be emptied.

Larvated (viable) STH ova counts were similar in the latrines with vs. without sand barriers, suggesting similar loading into the pit by infected individuals. Non-larvated ova counts were higher in pits with a sand barrier than pits without a sand barrier. This indicates that enveloping latrine pits with a sand layer helped contain helminth ova within the pits, allowing time for them to become non-viable and potentially reducing the spread of viable ova into the surrounding environment. The parent randomized trial that our study is nested in found that the sand barrier reduced bacterial transport into the surrounding shallow groundwater [15]. Helminth ova are larger than other enteric pathogens [17] and thus are more likely to be retained with an effective filtration method such as a sand barrier.

Sanitation improvements are considered key to sustainably control STH infections. While the principal approach to mitigating STH infections is mass drug administration, a systematic review and meta-analysis has shown that, 12 months post-treatment, the infection prevalence can revert to 94% of pre-treatment levels for *A*. *lumbricoides* and 82% for *T*. *trichiura* [26]. The frequent and widespread use of anthelmintic drugs may also result in the emergence of drug resistance, which would substantially reduce the effectiveness of the limited number of drugs currently available for treatment of STH infections [27]. Hence, environmental improvements that interrupt transmission cycles of STH may be critical to sustainably reduce the global burden of STH infections [28, 29]. A meta-analysis indicated that access to any latrine, improved or unimproved, was associated with reduced odds of STH infection [10], whereas another meta-analysis of cross-sectional studies on sanitation and STH infection found that the availability and use of improved sanitation facilities was associated with reduced odds of STH infection [11]. Pit latrines with a slab are more likely to be used and easier to clean, which could reduce the transfer of STH ova from the latrine to the household [12]. A recent randomized controlled trial that provided concrete-lined double-pit latrines in rural Bangladesh found reduced risk of infection with *T*. *trichiura* and hookworm but not *A*. *lumbricoides* among intervention recipients [30]. However, STH ova counts in courtyard soil were not affected by the intervention [13]. Our findings suggest that a sand barrier can help isolate STH ova from the environment when pit latrines are installed.

One limitation of this study is that we assessed the effect of sand barriers 18 and 24 months after latrine installation and cannot determine whether their effects would change over time or after pit emptying. We did not examine hookworm as our analytic method was not designed to catch fragile hookworm ova. We also did not investigate other types of enteric pathogens, which differ from helminths in their environmental fate and transport or whether the sand barrier can reduce leaching of chemical contaminants such as nitrogenous and carbon compounds. In addition, we relied on parameters measured inside the pit to assess decomposition and leaching but did not directly measure whether the sand barrier affected the presence or abundance of STH ova in underlying aquifers or adjacent surface water bodies. Similarly, we did not measure helminth ova in soils around the latrines to assess their dissemination from the pits. Additionally, we collected sludge from a single depth (1m). Therefore, we do not know if the intra-pit depth of fecal sludge sampling may impact the detection of helminth ova. While pathogens detected in sludge from different pit depths were not substantially different in a study in Malawi [31], latrine depth may impact the viability of STH ova as deeper layers of sludge represent less recent defecation. Finally, the study was conducted in one region and the area was selected by soil saturation and water table levels that represented the least favourable conditions for containment of pathogens. Findings could be different in areas with different hydrogeological features.

Our findings suggest that while sand barriers helped contain STH ova within the pits, latrines with sand barriers filled up more quickly, were emptied more frequently and had less complete decomposition. Pits were emptied into surface waters with no safety precautions. Efforts to increase access to safely managed sanitation as part of the Sustainable Development Goals should explore additional measures such as double-pit designs and promotion of safe sludge disposal practices to overcome these shortcomings. Further research is also needed on latrine technologies that can both effectively isolate pathogens from the environment and achieve rapid decomposition.

## Supporting information

S1 Text Methods for pit latrine and monitoring well installation. (PDF)

S2 Text Standard Operation Procedure (SOP) of fecal sludge sampling. (PDF)
