# Supplementary material for: Higher helminth ova counts and incomplete decomposition in sand-enveloped latrine pits in a coastal sub-district of Bangladesh
Source: PLoS Negl Trop Dis. 2022 Jun 23;16(6):e0010495. doi: 10.1371/journal.pntd.0010495 (PMC9223371; doi:10.1371/journal.pntd.0010495)
Supplement: S1 Text — (PDF) [file pntd.0010495.s001.pdf]

Supporting information

S1 Text. Methods for pit latrine and monitoring well installation

WASHplus selected 68 sites to study the effectiveness of sand envelopment on mitigating groundwater pollution from pour-flush pit latrines in Golachipa, Bangladesh. The superstructure for all 68 latrines was installed following the WASHplus design furnished by WaterAid. The characteristics of offset pits were modified slightly from the WASHplus design to include a 50 cm sand barrier in the experimental latrines and no sand barrier in the control group.

This document describes the construction process that vendors contracted by the local nongovernmental organization South Asia Partnership-Bangladesh, in coordination with icddr,b staff, followed to construct the latrine pits used in the study.

General procedures

icddr,b staff supervising the work confirmed that the site conforms to the three selection criteria outlined in the technical protocol (e.g., four to 10 members per household, at least 10 steps distance from an existing latrine and not close to a body of water). icddr,b field staff used a hand auger to dig a hole at the site identified for the offset pit. The hole extended until the groundwater table was reached. icddr,b staff measured the depth to groundwater table from the ground surface using a measuring tape or electronic probe (probe was preferable).Once the depth to groundwater table was determined, icddr,b staff calculated the depth to the bottom of the pit considering the depth to groundwater measurement. This ensured the bottom concrete ring in the pit sits in the unsaturated soil (on the day of construction) above the groundwater table. The number of concrete rings used at each pit was a function of the depth to bottom of pit. The study

team expected that all pits had between three and five rings depending on the characteristics at the site. In keeping with the WASHplus latrine design, one ring was placed directly under the pan, which protruded above the ground surface to give the slab a 1-foot-high pedestal for water seal and PVC pipe to bend accordingly from the pan.

The study team tried to follow a standard design for all the study latrines, but if the groundwater level was higher in some locations, then the number of pit rings was reduced to maintain the proposed distance with the bottom of the pit. The field research assistants (FRAs) informed the investigators before modifying the design and reducing the number of pit rings. After excavating the pit to the specified depth, the FRA collected a 50cm soil sample from the center point of the pit bottom for all 68 latrine sites. The sample was collected using a pre-labelled 2-foot-long and 1.5-inch diameter PVC pipe. After collection, the pipe was wrapped with aluminum foil to prevent spillage. The FRA marked the sample with date and household ID. The soil removed by during sample collection was replaced by fill soil removed during the excavation.

Latrines without sand barrier or control latrines

Of 68 study latrines, 34 pits did not have a sand barrier. At these sites, the vendor excavated a 75 cm diameter hole to the desired depth specified by the icddr, b supervisor. The depth of the pit was above the water table on the day of construction. A varying number of concrete rings were placed into the pit, following one ring extending to just above the surface. Concrete rings was stacked on top of each other and not sealed with mortar. Care was taken to minimize the annulus between the outer edge of the concrete rings and soil wall. There was no other modification in the existing WASHplus design.

Latrines with sand barrier or intervention latrines

Thirty-four pits had a 50 cm sand barrier in the bottom and surrounding the pit. At these sites, the vendor excavated a 1.75 m diameter hole to the desired depth specified by the icddr, b supervisor. The depth of the pit was above the water table on the day of construction. Once excavation was completed, coarse sand was added to the bottom of the pit and compacted every 20cm using a handheld rammer. This process was continued until the sand layer was 50cm thick across the entire base of the pit. The study team used a marked stick in the center to measure the 50 cm thickness of the soil. After reaching the desired thickness, the stick was removed, and the center was compacted to fill up the empty space. Vendors then placed the bottom concrete ring into the center of the pit, checking that the annulus between the concrete wall and the sides of the pit was equidistant on all sides. Sand was poured around the ring and compacted every 20 cm until the sand reaches the top of the first concrete ring. This process was repeated with the next ring until the rings and sand envelope reach the surface. A layer of 20 cm stone aggregate was placed into the pit, resting on top of the sand layer at the bottom of the pit.
