# Supplementary material for: Higher helminth ova counts and incomplete decomposition in sand-enveloped latrine pits in a coastal sub-district of Bangladesh
Source: PLoS Negl Trop Dis. 2022 Jun 23;16(6):e0010495. doi: 10.1371/journal.pntd.0010495 (PMC9223371; doi:10.1371/journal.pntd.0010495)
Supplement: S2 Text — (PDF) [file pntd.0010495.s002.pdf]

Supporting information

S2 Text. Standard Operation Procedure (SOP) of fecal sludge sampling

Lab staff put on gloves and a lab coat before preparing the sample. Then they separated the sludge samples into two aliquots: (1) Soil-transmitted helminth contamination (15 g) and (2) Moisture content (5 g). Lab staff shacked the sludge samples thoroughly to mix it before removing aliquots. A 15 g of sludge was measured and placed into a 50 mL centrifuge tube. After that added 1% 7X up to the 35 mL line and hand-shaken vigorously for 3 minutes. Next, rinsed sides and cap until 7X was up to 40 mL line. The mixture was soaked overnight, then hand-shaken for 10 minutes and vortexed on 2000 rpm for 15 seconds to dislodge STH ova from particles. Then rinsed with additional 1% 7X three times. After that the sieve was rinsed and brush cleaned with detergent in between samples. The supernatant was left to settle for 2 hours and washed everything from previous steps. Then aspirated with a hand pump or vacuum without disturbing the residue at the bottom. Each sample was poured into two 50 mL centrifuge tubes and rinsed beaker with 1% 7X thoroughly and distribute into centrifuge tubes. Approximately 40 mL of 1% 7X solution was added to the precipitate and the solution was centrifuged at 1000 g for 10 minutes and balanced centrifuge within 0.5 g; the supernatant was discarded.

Next, 5 mL of zinc sulphate flotation solution (1.25 specific gravity) was added to the precipitate, vortexed for 30 seconds, centrifuged at 1000 g for 5 minutes and the supernatant was saved; this procedure was conducted a total of three times. The combined supernatant from the

three flotation steps was filtered through a 500-mesh sieve to capture STH ova. The sieve was rinsed into a Falcon tube using distilled water into a 50 mL centrifuge tube. Next added distilled water up to 40 mL line depending on how full the tubes already are. The rinse water was centrifuged at 1000 g for 5 minutes and balanced centrifuge within 0.5 g. The supernatant was removed with a pipette until there was 1 mL left at the bottom of the tube. Next, 25 mL of 0.1 N sulfuric acid solutions was added to the tube. The tube was capped loosely (not airtight) and incubated at 28° C for 28 days to allow viable ova to develop larvae.

After 28 days, tubes were removed from incubator to count eggs and counted on the same day. At the end of the incubation period, the solution was centrifuged at 1000 g for 3 min and aspirated using a pipette to aspirate the solution to a final volume of 1 mL. The 1 mL solution was transferred using a new, disposable 1 mL pipette to a Sedgewick-Rafter slide and examined under the microscope for *A. lumbricoides* and *T. trichiura* ova using a visual identification chart to distinguish the type of ova and whether it was larvated or non-larvated. The numbers of larvated and non-larvated ova for each species were recorded separately to differentiate viable and non-viable ova. An additional 5 g soil aliquot was oven-dried over night to determine moisture content and dry weight.

For quality assurance and quality control, 10% of samples were processed in replicate, and a laboratory blank was processed once every other day by repeating the protocol without a soil sample. 10% of samples were counted by two independent analysts to assess interrater reliability. Additionally, for each sample, lab technicians took a picture of the first occurrence of each type

of ova (larvated *A. lumbricoides*, non-larvated *A. lumbricoides* etc.); the pictures were reviewed for accuracy of categorization by study investigators.
